# AIDA-1 Moves out of the Postsynaptic Density Core under Excitatory Conditions

**DOI:** 10.1371/journal.pone.0137216

**Published:** 2015-09-10

**Authors:** Ayse Dosemeci, Dana Toy, Thomas S. Reese, Jung-Hwa Tao-Cheng

**Affiliations:** 1 Laboratory of Neurobiology, National Institute of Neurological Disorders and Stroke, National Institutes of Health, Bethesda, Maryland, United States of America; 2 EM Facility, National Institute of Neurological Disorders and Stroke, National Institutes of Health, Bethesda, Maryland, United States of America; University of Sydney, AUSTRALIA

## Abstract

AIDA-1 is highly enriched in postsynaptic density (PSD) fractions and is considered a major component of the PSD complex. In the present study, immunogold electron microscopy was applied to determine localization as well as the activity-induced redistribution of AIDA-1 at the PSD using two antibodies that recognize two different epitopes. In cultured rat hippocampal neurons under basal conditions, immunogold label for AIDA-1 is mostly located within the dense core of the PSD, with a median distance of ~30 nm from the postsynaptic membrane. Under excitatory conditions, such as depolarization with high K^+^ (90 mM, 2 min) or application of NMDA (50 μM, 2 min), AIDA-1 label density at the PSD core is reduced to 40% of controls and the median distance of label from the postsynaptic membrane increases to ~55 nm. The effect of excitatory conditions on the postsynaptic distribution of AIDA-1 is reversed within 30 minutes after returning to control conditions. The reversible removal of AIDA-1 from the PSD core under excitatory conditions is similar to the redistribution of another abundant PSD protein, SynGAP. Both SynGAP-alpha1 and AIDA-1 are known to bind PSD-95. Activity-induced transient translocation of these abundant proteins from the PSD core could promote structural flexibility, vacate sites on PSD-95 for the insertion of other components and thus may create a window for synaptic modification.

## Introduction

The postsynaptic density (PSD), a large protein complex lining the postsynaptic membrane, contains an organized array of receptors and signaling molecules. The PSD scaffold consists of several proteins of the MAGUK, GKAP, Shank and Homer families with specialized protein-protein association domains that anchor and organize components of the PSD [2].

AIDA-1 (amyloid-beta protein precursor intracellular domain associated protein 1, also known as ankyrin repeat and sterile alpha motif domain-containing protein 1B) is another family of proteins with multiple protein-protein association domains present at the PSD. Short forms of AIDA-1 (AIDA-1d and AIDA-1e, Q8BZM2) are highly enriched in PSD fractions from the brain [3], [4]. Specific localization of AIDA-1 at PSDs in intact neurons has been verified by immuno-electron microscopy [5]. NMDA-induced AIDA-1 translocation from the synapse to the nucleus has been proposed to regulate protein synthesis [3]. A recent study describes an additional function of AIDA-1 in the regulation of NMDA receptor subunit GluN2B [6].

**Fig 1:**
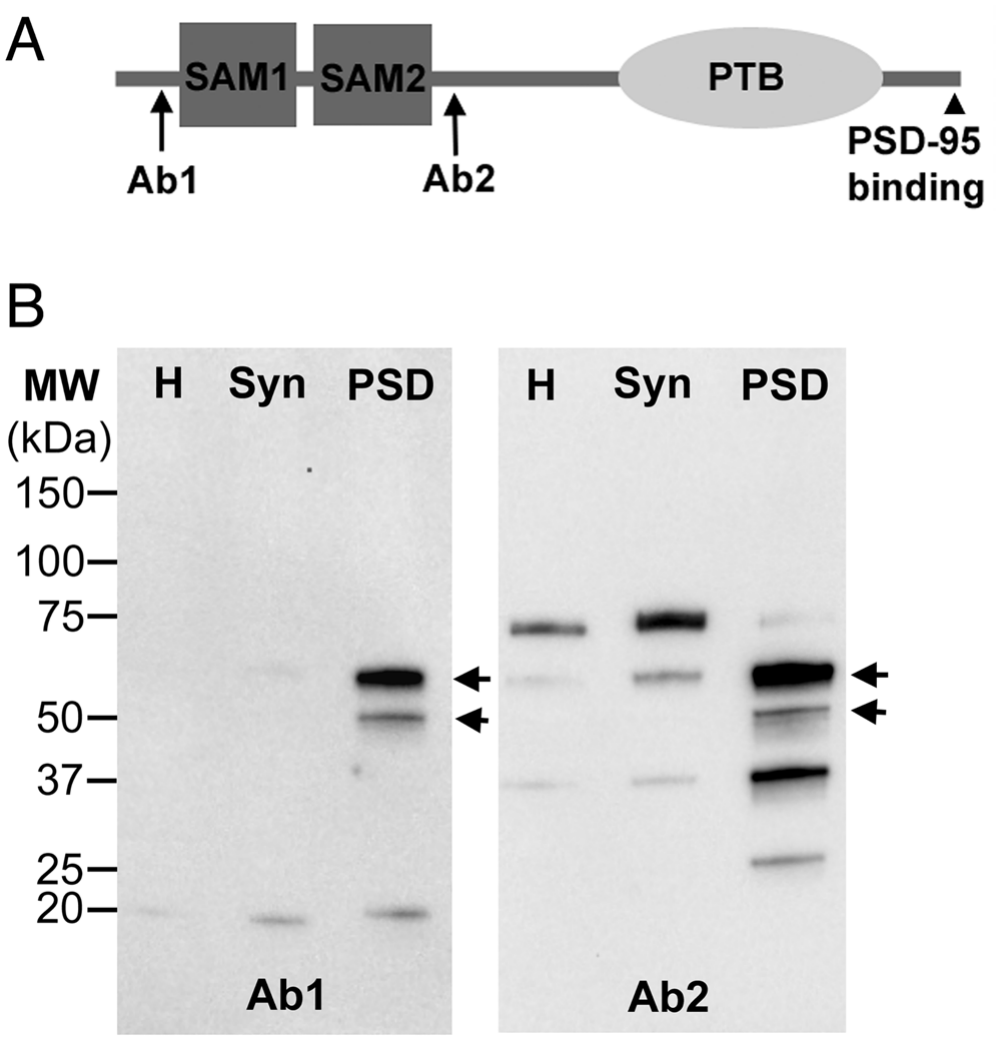
**(A)** The short AIDA-1 proteins (P0C6S7-2 or AIDA-1e, P0C6S7-3 or AIDA-1d in rat as designated in UniProt) contain two SAM domains and a PTB domain, but lack the characteristic ankyrin repeats of the long form (P0C6S7-1, 139 kDa). Antibody 1 (Ab 1) and antibody 2 (Ab 2) were raised against peptides corresponding to epitopes near the N-terminal and near SAM2 domain respectively (arrows). PSD-95 binding is at the C-terminal (arrowhead). **(B)** Western immunoblots using the two antibodies. Homogenate (H) and synaptosome (Syn) fractions containing 10 μg protein and PSD fractions containing 5μg protein were applied to each lane. Positions of ~50 kDa and ~60 kDa bands recognized by both antibodies are shown by arrows.

AIDA-1 contains two sterile alpha motif (SAM) domains and a phosphotyrosine-binding (PTB) domain and, through its C-terminal, associates with PSD-95 [3](Fig 1A). SAM is a protein-protein interaction domain, also found in the Shank family of proteins. SAM domains from Shank3 were shown to self-assemble into large sheets of helical fibers [7], suggesting that SAM-containing proteins may associate with each other at the PSD. PTB region of AIDA-1 binds to the intracellular domain of amyloid-beta protein precursor APP [8] and may anchor this protein at the synaptic cleft. The stoichiometry of AIDA-1 to GKAP proteins and PSD-95 at the PSD has recently been estimated as 1:1:2 [9]. The high abundance of AIDA-1 at the PSD, as well as its capacity to bind multiple synaptic components suggest a role in the structural organization of the PSD.

Activity-induced changes in the number and localization of proteins at the PSD complex are thought to underlie changes in synaptic strength. We have previously shown that PSDs exhibit molecular re-organization during synaptic activity. Under excitatory conditions, more CaMKII, Shank and CYLD accumulate within the deeper zone of the PSD, contiguous to the electron dense core [10], [11], [12], while another abundant PSD protein, SynGAP, moves out of the PSD core [13]. In contrast, other components, such as PSD-95 and GKAP retain their positioning under excitatory conditions [13], [14]. In the present study, we explored possible redistribution of AIDA-1 at the PSD under excitatory conditions.

## Materials and Methods

### Antibodies for AIDA-1

A polyclonal rabbit antibody was produced by Affinity Bioreagents against the peptide LKRFPVHPVTGPR, corresponding to the N-terminal of Q8BZM2 (antibody1). A second polyclonal rabbit antibody raised against a polypeptide with a sequence RLHDDPPQKPPRSIT corresponding to residues 946–960 of Q7Z6G8 (Human AIDA-1) was obtained from Zymed.

### Subcellular fractionation and Western immunoblotting

Brains from young adult or mature adult Sprague Dawley rats of either sex were collected and frozen in liquid nitrogen within 2 min of decapitation by either Pel-Freez Biologicals (Rogers, AR) or Rockland (Gilbertsville, PA) and shipped on dry ice. Brains were thawed by one min immersion in isotonic sucrose at 37°C and dissected immediately. Cerebral cortices were homogenized in isotonic sucrose. Synaptosome and PSD fractions were obtained as described previously [15]. Samples were resolved by SDS-PAGE using 4–15% gradient Mini PROTEAN TGX precast polyacrylamide gels (Bio-Rad), transferred to PVDF membranes using the Trans-Blot Turbo Transfer System (Bio-Rad), blocked and incubated with primary and then secondary antibodies. Immunoblots were visualized by chemiluminescence (Thermo Scientific).

### Preparation and treatment of dissociated hippocampal cultures

The animal protocol was approved by the National Institute of Neurological Disorders and Stroke/ National Institute of Deafness and Communication Disorders/National Center for Complementary and Integrative Health Animal Use and Care Committee and conforms to NIH guidelines. Hippocampi from 20–21 day embryonic Sprague-Dawley rats were dissociated and grown on a feeder layer of glial cells for 3 weeks. During experiments, culture dishes were placed on a floating platform in a water bath maintained at 37°C. Control incubation medium contained 124 mM NaCl, 2 mM KCl, 1.24 mM KH_2_PO_4_, 1.3 mM MgCl_2_, 2.5 mM CaCl_2_, 30 mM glucose in 25 mM HEPES at pH 7.4. High K^+^ solution contained 90 mM KCl and the osmolarity was maintained by reducing NaCl concentration. Cell cultures were washed with control medium and then treated for 2 min with control, high K^+^, or NMDA (50 μM NMDA in control medium) media. For recovery experiments, samples were treated with high K^+^ or NMDA for 2 min and then washed with control medium (5 times within 2 min), then further incubated in control medium for a total of 30 min prior to fixation. Cells were fixed with 4% paraformaldehyde (EMS, Fort Washington, PA) in PBS for 25–35 min, and thoroughly washed in PBS and stored at 4°C before immunolabeling.

### Pre-embedding immunogold labeling and electron microscopy

Unless otherwise indicated the protocol was carried out at room temperature. Samples were permeablized and blocked with a solution of 0.1% saponin and 5% normal goat serum in PBS for 30–60 min. Subsequently, samples were incubated with primary and then secondary antibodies (Nanogold, Nanoprobes, Yaphand, NY) for 1 hr each, fixed with 2% glutaraldehyde in PBS for 30 min and stored at 4°C. Samples were subsequently washed in deionized water, silver enhanced (HQ kit, Nanoprobes), treated with 0.2% osmium tetroxide in 0.1 M phosphate buffer at pH 7.4 for 30 min on ice, treated with 0.25% uranyl acetate in acetate buffer at pH 5.0 for 1 hr at 4°C, dehydrated in graded ethanols, and embedded in epoxy resin.

### Morphometry

Asymmetric excitatory synapses were identified by their characteristic dense material underneath the postsynaptic membrane, clusters of synaptic vesicles in the presynaptic terminal, and rigidly apposed pre- and post-synaptic membranes forming a synaptic cleft. At least five randomly selected grid openings were examined for each sample. Every cross-sectioned synaptic profile labeled for AIDA-1 was photographed with a bottom-mounted digital CCD camera (AMT XR-100, Danvers, MA, USA) for morphometry.

**Fig 2:**
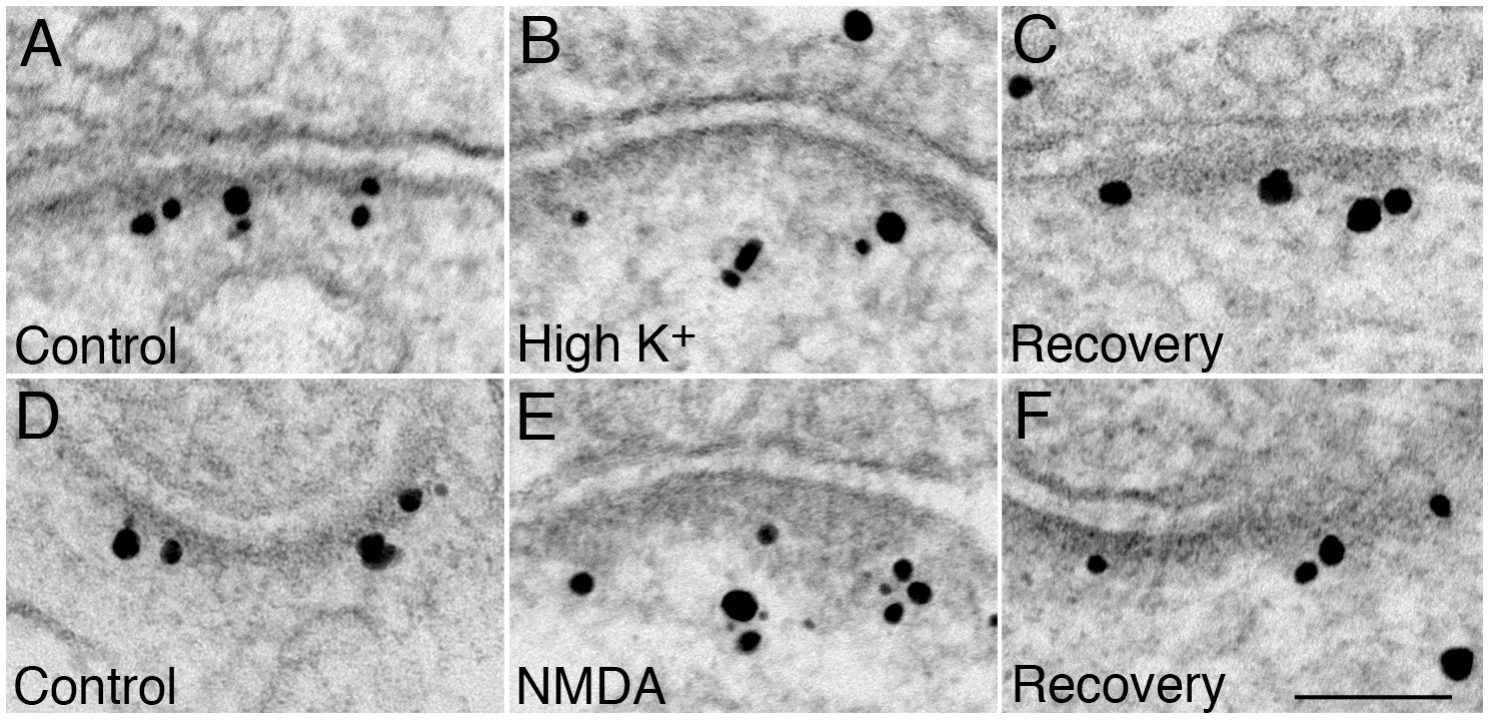
Electron micrographs of asymmetric synapses in dissociated hippocampal neuronal cultures immunogold labeled for AIDA-1. Antibody 1 was used in samples depicted in the upper row (A-C) and antibody 2 was used in samples depicted in the lower row (D-F). Dark grains of heterogeneous size are silver enhanced gold particles representing AIDA-1 label. Cultures were treated with either control medium (A and D), high K^+^ (B and C) or NMDA (E and F) for 2 min, and then either fixed immediately (A,B,D,E) or let to recover for 30 min in control medium (C and F) before fixing. Scale bar = 0.1 μm.

The measurement area of the PSD complex was outlined by the postsynaptic membrane, two parallel lines dropped perpendicular to the postsynaptic membrane, and an arbitrary border 120 nm deep to the postsynaptic membrane (cf. Yang., 2011). The PSD complex was further divided into two compartments: (1) the PSD core, an area adjacent to the postsynaptic membrane containing characteristic dense material, and (2) the PSD pallium, or the contiguous network (Yang et al 2011), extending deeper into the cytoplasm. The PSD pallium cannot always be directly visualized by techniques used for conventional EM, but can be inferred from characteristic immunolabeling for Shank and Homer proteins [11], [16]. The boundary for counting gold particles within the PSD core was set at 40 nm from the postsynaptic membrane, encompassing ~30 nm thick electron dense zone with a 10 nm extension to allow for antibody span between epitope and gold particle. The counting area for the contiguous PSD pallium extended from 40 to 120 nm deep from the postsynaptic membrane. Every particle within the two designated areas of the PSD complex was counted and the sum was divided by the length of the PSD as an index of labeling density (number of particles / μm length of PSD). The raw data is compiled in S1 Table. Due to variations in labeling efficiency among experiments, values for different experimental conditions were normalized to controls within each experiment for comparison. For the same reason, electron micrographs in Fig 2 were selected from the same experiment.

To assess the laminar distribution of AIDA-1 at PSDs with respect to the postsynaptic membrane, the distance from the center of the gold particle to the outer edge of the postsynaptic membrane was measured for every particle in the marked PSD complex area. Because the laminar distribution of AIDA-1 under stimulated conditions was typically skewed, values for median instead of mean were used for a nonparametric statistical test (Wilcoxon test; KaleidaGraph, Synergy Software, Reading, PA).

## Results

Western blots of subcellular fractions from rat brains were probed with two antibodies against distinct epitopes on AIDA-1 (Fig 1A). Two electrophoretic bands of ~50 kDa and ~60 kDa are recognized by both antibodies (Fig 1B, arrows). Both bands are highly enriched in PSD fractions compared to parent homogenate and synaptosome fractions (Fig 1B). Two bands of similar molecular weights have previously been identified in PSD fractions as AIDA-1d and AIDA-1e by Jordan et al [3] using different antibodies. The specificity of antibody 2, which recognizes additional bands, has been further previously verified [5]. A 75 kDa band recognized by antibody 2 is enriched in the synaptosome fraction but does not co-purify with PSDs.

Pre-embedding immunoEM with either antibody 1 or antibody 2 show selective labeling of PSDs in dissociated hippocampal neuronal cultures (Fig 2). Under basal conditions, AIDA-1 label is close to the postsynaptic membrane, mostly within the dense core of the PSD (Fig 2A and 2D). Under excitatory conditions, depolarization by exposure to high potassium (90 mM K^+^ for 2 min) or NMDA treatment (50μM for 2 min), AIDA-1 label becomes localized further away from the postsynaptic membrane (Fig 2B and 2E). These effects are reversed within 30 min after the cessation of excitatory conditions (Fig 2C and 2F).

Results from multiple immunoEM experiments using either antibody 1 or antibody 2 were quantified. The densities of AIDA-1 label at the PSD core (area extending 40 nm deep from the postsynaptic membrane) were determined for individual synapses, and the values of mean ± SEM from different experiments were listed in S1 Table. Average label densities for each condition (control, NMDA or high K^+^ treatment and 30 min recovery) were expressed as a percentage of control values (Table 1) for easy comparison. Results from three experiments with antibody 1 and two experiments with antibody 2 consistently showed a decrease in the density of label for AIDA-1 at the PSD core under excitatory conditions, with statistical significance reached in all experiments with K^+^ and four out of five experiments with NMDA (one-way ANOVA). The mean AIDA-1 label density at the PSD core following either high K^+^ or NMDA treatments decreased on average to ~40% of controls (five experiments each, Table 1).

**Table 1:** AIDA-1 label density at the PSD core decreases under excitatory conditions and returns to basal levels within 30 min after cessation of the stimulus.

Density of label in the PSD core normalized to control (n = number of PSDs)						
	Experiment #	Control	K^+^	K^+^ Recovery	NMDA	NMDA Recovery
Ab1	1	100% (31)	51% (25)	106% (14)	27% (14)	84% (24)
	2	100% (43)	42% (50)	-	58% (36)	-
	3	100% (38)	29% (29)	74% (35)	72% (39)	80% (32)
Ab2	4	100% (31)	24% (25)	115% (35)	-	-
	5	100% (27)	44% (27)	-	19% (19)	-
	6	100% (44)	-	-	32% (31)	107% (45)
**Mean±SEM**		**100%**	**38±5.0%**	**98±12.4%**	**42±10.0%**	**90±8.4%**

Statistical significance evaluated by one-way ANOVA with Tukey’s post test. Control vs. K^+^: p<0.0001; K^+^ vs. K^+^ Recovery: p<0.001; Control vs. K+ Recovery: non-significant; Control vs. NMDA: p<0.0005; NMDA vs. NMDA Recovery: p<0.005; Control vs. NMDA Recovery: non-significant.

Parallel measurements carried out in the contiguous zone, the PSD pallium (area extending from 40 to 120 nm deep from the postsynaptic membrane), showed a concomitant increase in AIDA-1 label density (S1 Table), which was consistent and statistically significant (one-way ANOVA) in all experiments. Average label densities for each condition were expressed as a percentage of control values (Table 2). Within 30 min following the removal of high potassium- or NMDA-containing media (recovery), AIDA-1 label densities in both compartments of the PSD became statistically indistinguishable from control values in all experiments (Tables 1 & 2).

**Table 2:** AIDA-1 label density at the PSD pallium increases under excitatory conditions and returns to basal levels within 30 min after cessation of the stimulus.

Density of label in PSD pallium normalized to control (n = number of PSDs)						
	Experiment #	Control	K^+^	K^+^ Recovery	NMDA	NMDA Recovery
Ab1	1	100% (31)	273% (25)	115% (14)	270% (14)	60% (24)
	2	100% (43)	232% (50)	-	458% (36)	-
	3	100% (38)	176% (29)	63% (35)	432% (39)	116% (32)
Ab2	4	100% (31)	289% (25)	114% (35)	-	-
	5	100% (27)	181% (27)	-	297% (19)	-
	6	100% (44)	-	-	283% (31)	114% (45)
**Mean+SEM**		**100%**	**230±23.1%**	**97±17.2%**	**348±40.0%**	**97±18.3%**

Statistical significance evaluated by one-way ANOVA with Tukey’s post test. p<0.05 for the following comparisons: Control vs. K^+^; K^+^ vs. K^+^ Recovery. Control vs. K^+^ Recovery: non-significant. p<0.0001 for the following comparisons: Control vs. NMDA; NMDA vs. NMDA Recovery. Control vs. NMDA Recovery: non-significant.

**Fig 3:**
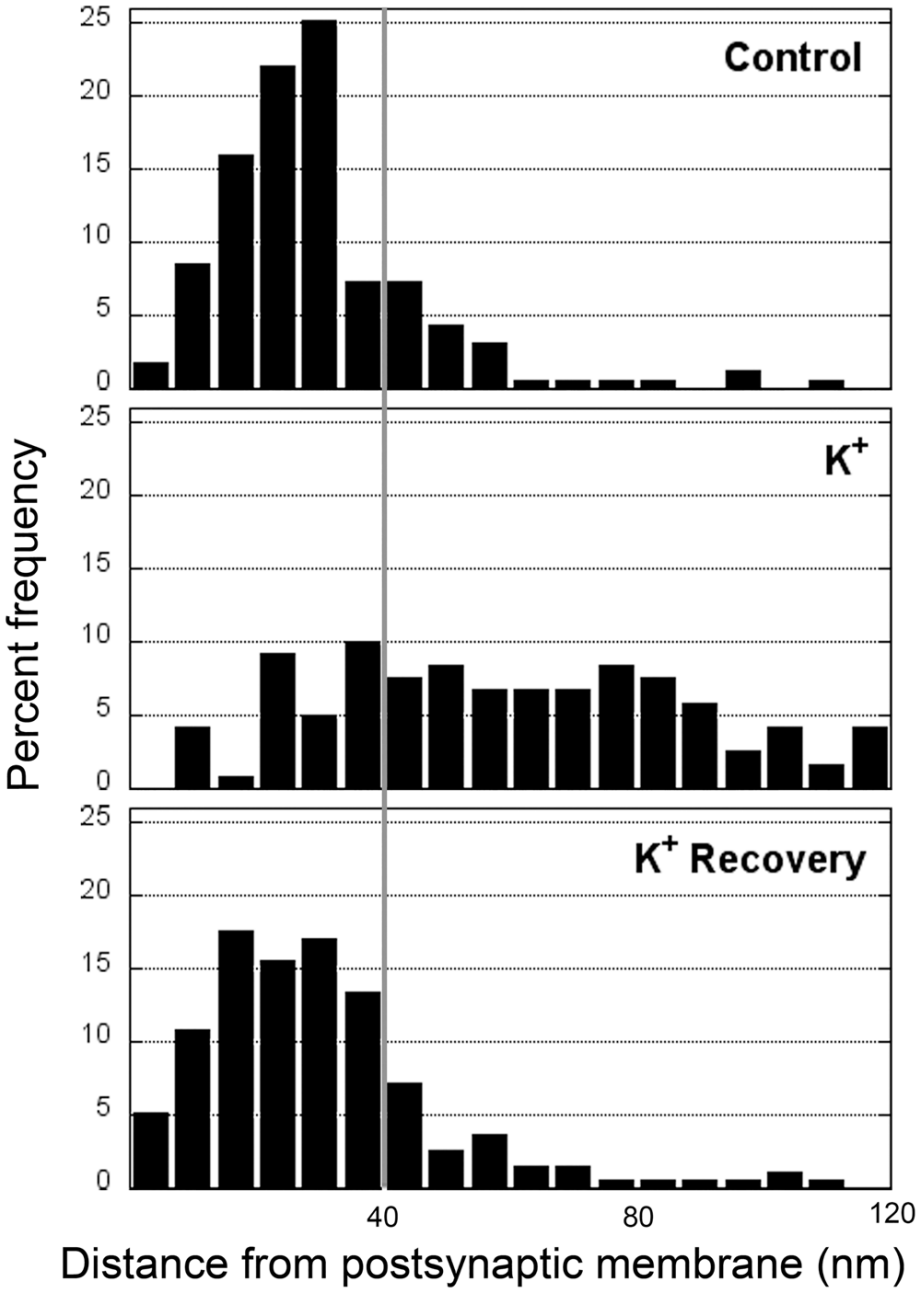
The distribution of AIDA-1 at the PSD complex is regulated by activity. Histograms from a representative experiment depicting the distribution of AIDA-1 label, within an area extending 120 nm deep from the postsynaptic membrane. Distances of gold particles (AIDA-1 label) from the postsynaptic membrane were measured in control samples, in depolarized samples (K^+^) and in samples were let to recover for 30 min in control medium after depolarization (K^+^ recovery). The vertical line depicts assigned threshold separating the PSD core from the contiguous zone (PSD pallium).

The results described above indicate a decrease in the amount of AIDA-1 at the PSD core under excitatory conditions. In order to obtain a more detailed view of the redistribution of AIDA-1 under excitatory conditions, the distance of each individual gold particle from the postsynaptic membrane was measured within the entire zone of the PSD complex, an area extending 120 nm deep from the postsynaptic membrane and graphed into histograms. Fig 3 illustrates results from a representative experiment. The increase in the median distance of gold particles from the membrane upon treatment with high potassium or NMDA was consistent and statistically significant in all experiments (Wilcoxon test). Compilation of the measurements from all experiments (Table 3) show that the typical median distance of AIDA-1 label from the postsynaptic membrane under basal conditions is ~30 nm. Upon depolarization with high K^+^ or treatment with NMDA, the typical median distances of label from the postsynaptic membrane increase to 54 nm and 59 nm, respectively. Following cessation of excitatory stimuli, both depolarization and NMDA-induced shifts return to control values within 30 min.

**Table 3:** Median distance of AIDA-1 label from the postsynaptic membrane increases under excitatory conditions and returns to basal values within 30 min after cessation of the stimulus.

Median distance of label from postsynaptic membrane in nm (n = number of gold particles; number of PSDs)						
	Exp #	Control	K^+^	K^+^ Recovery	NMDA	NMDA Recovery
Ab1	1	**30.0** (154; 31)	**56.7** (159; 25)	**33.3** (99; 14)	**63.3** (95; 14)	**26.7** (84; 24)
	2	**30.0** (211; 43)	**53.3** (220; 50)	-	**56.7** (296; 36)	-
	3	**30.0** (228; 38)	**56.7** (122; 29)	**26.7** (129; 35)	**46.7** (312; 39)	**30.0** (168; 32)
Ab2	4	**30.0** (163; 31)	**60.0** (119; 25)	**30.0** (193; 35)	-	-
	5	**33.3** (137; 27)	**43.3** (93; 27)	-	**70.0** (89; 19)	-
	6	**26.7** (177; 44)	-	-	**56.7** (141; 31)	**30.0** (199; 43)
**Mean±SEM**		**30.0±0.9**	**54.0±2.9**	**30.0±1.9**	**58.7±3.9**	**28.9±1.1**

Statistical significance evaluated by one-way ANOVA with Tukey’s post test. p< 0.0001 for all of the following comparisons: Control vs. K^+^; K^+^ vs. K^+^ Recovery; Control vs. NMDA and NMDA vs. NMDA Recovery. Differences between Control vs. K^+^ recovery and Control vs. NMDA Recovery were statistically non-significant.

## Discussion

Two short isoforms of AIDA-1 are found enriched in the PSD fraction, in agreement with a previous study using different antibodies [3]. AIDA-1 label is selectively localized at the PSD by pre-embedding immunoEM. Under basal conditions AIDA-1 label is mostly concentrated within the PSD core, defined as a 40 nm wide area adjacent to the postsynaptic membrane, consistent with the previously described localization in adult rat brain by post-embedding immunoEM techniques [5].

Antibody 1 and antibody 2 recognize epitopes near the N-terminal and near the middle, respectively, of the AIDA-1 short isoforms (Fig 1A) and both yielded distributions of label with ~30 nm median distance from the postsynaptic membrane. The distributions of label with the two AIDA-1 antibodies at the PSD are similar to the distribution of label observed previously with an antibody against PSD-95 that recognizes an epitope close to its binding sites to the C-terminal of AIDA-1 [13]. Thus, it can be envisaged that when AIDA-1 is anchored to PSD-95, it must be positioned with its N-terminus, mid section and C-terminus aligned roughly parallel to the postsynaptic membrane.

Upon depolarization with high K^+^ or treatment with NMDA, a major fraction (~60%) of AIDA-1 label moves out of the PSD core. Both treatments result in a significant increase in the median distance of label from the postsynaptic membrane. The amount of the shift promoted by either high K^+^ or NMDA shows large variations from experiment to experiment. The variability may be due to the graded nature of translocation, variable diffusion properties of the protein once outside the PSD core, or other differences between batches of hippocampal cultures. Within 30 min after cessation of excitatory conditions, AIDA-1 distribution at the PSD becomes indistinguishable from that in control samples. Altogether, immunoEM observations indicate temporary displacement of AIDA-1 out of the PSD core under excitatory conditions.

The reversible translocation of AIDA-1 described in the present study is surprisingly similar to that previously observed for SynGAP, another abundant PSD protein. Like AIDA-1, SynGAP moves out of the PSD core during depolarization or treatment with NMDA and becomes once again concentrated at the core upon cessation of excitatory conditions [13] [1]. Both of these proteins associate with PSD-95 at its PDZ domains [17], [3]), sites also used for the association of glutamate receptors. It is probable that AIDA-1, as well as SynGAP, have to detach from PSD-95 to move away from the PSD core. Transient increase in the concentration of AIDA-1 at the pallium could be indicative of alternative attachment sites for the protein in that location. Presence of additional attachment sites for AIDA-1 would be in agreement with the observations of Mulholland et. al. [18] which indicate that PSD-95 is not necessary for synaptic localization of AIDA-1.

It is generally accepted that insertion or removal of receptors from the synapse underlies modifications of synaptic strength. However, from the perspective of the structural biologist, it has remained a dilemma of how this can be achieved within the rigidly ordered and crowded array of proteins in the PSD. The present study on activity-induced translocation of AIDA-1, together with a previous study showing a similar movement of SynGAP, offers a potential mechanism through structural re-organization. SynGAP and AIDA-1 are among the most abundant components of the PSD, where copy numbers of SynGAP exceed those of PSD-95 [19] and copy numbers of AIDA-1 are about half those of PSD-95 [9]. We hypothesize that activity-induced transient removal of these abundant PSD-95-associated proteins from the PSD core would render the core more available for protein trafficking and also vacate binding sites on PSD-95, thus providing a window for reorganizing receptors.

## Supporting Information

S1 Table AIDA-1 label densities at the PSD core and PSD pallium expressed as number of gold particles per μm PSD. (PDF)
