# Supplementary material for: AIDA-1 Moves out of the Postsynaptic Density Core under Excitatory Conditions
Source: PLoS One. 2015 Sep 10;10(9):e0137216. doi: 10.1371/journal.pone.0137216 (PMC4565644; doi:10.1371/journal.pone.0137216)
Supplement: S1 Table — (PDF) [file pone.0137216.s001.pdf]

S1 Table: AIDA-1 label density decreases at the PSD core and increases at the PSD pallium under excitatory conditions, and returns to basal levels within 30 min after cessation of the stimulus.

Density of label expressed as number of gold particles/μm PSD (n= number of PSDs)							
	**Exp #**		**Control**	**K^+**	**K^+ Recovery**	**NMDA**	**NMDA Recovery**
**Ab 1**	**Exp 1**	**core**	11.8 ± 1.2 (31)	6.0 ±1.0 (25)	12.5 ± 2.2 (14)	3.2 ± 0.9 (14)	9.9 ±1.3 (24)
		**pallium**	4.0 ± 0.7	10.9 ± 1.6	4.6 ± 1.2	10.8 ± 2.4	2.4 ± 0.8
	**Exp 2**	**core**	10.8 ± 1.1 (43)	4.5 ± 0.6 (50)	-	6.3 ± 1.0 (36)	-
		**pallium**	3.1 ± 0.6	7.2 ± 0.9	-	14.2 ± 1.5	-
	**Exp 3**	**core**	13.3 ± 1.2 (38)	3.9 ± 1.1 (29)	9.8 ± 0.9 (35)	9.6 ±1.8 (39)	10.6 ± 1.2 (32)
		**pallium**	3.8 ± 0.8	6.7 ± 0.7	2.4 ± 0.7	16.4 ± 5.1	4.4 ± 1.0
**Ab 2**	**Exp 1**	**core**	13.3 ± 1.5 (31)	3.2 ± 0.7 (25)	15.3 ± 1.9 (35)	-	-
		**pallium**	2.8 ± 0.8	8.1 ± 0.9	3.2 ± 0.8	-	-
	**Exp 2**	**core**	10.1 ± 1.0 (27)	4.4 ± 0.9 (27)	-	1.9 ± 0.4 (19)	-
		**pallium**	3.1 ± 0.8	5.6 ± 0.9	-	9.2 ± 1.2	-
	**Exp 3**	**core**	11.1 ± 1.3 (44)	-	-	3.5 ± 0.7 (31)	11.9 ± 1.3 (45)
		**pallium**	2.9 ± 0.8	-	-	8.2 ± 1.3	3.3 ± 0.9
**Combined (mean ± SEM)**		**core**	**11.7 ± 0.5**	**4.4 ± 0.5**	**12.5 ± 1.6**	**4.9 ± 1.4**	**10.8 ± 0.6**
		**pallium**	**3.3 ± 0.2**	**7.7 ± 0.9**	**3.4 ± 0.6**	**11.8 ± 1.5**	**3.4 ± 0.6**
